# Numerical Study on 4-1 Coal Seam of Xiaoming Mine in Ascending Mining

**DOI:** 10.1155/2015/516095

**Published:** 2015-03-17

**Authors:** Lan Tianwei, Zhang Hongwei, Li Sheng, Han Jun, Song Weihua, A. C. Batugin, Tang Guoshui

**Affiliations:** ^1^School of Mines, Liaoning Technical University, Fuxin 123000, China; ^2^State Key Laboratory of Coal Resources and Mine Safety, China University of Mining and Technology, Xuzhou 211116, China; ^3^Key Laboratory of Mine Thermo-Motive Disaster and Prevention, Ministry of Education of Liaoning Technical University, Fuxin 123000, China; ^4^Center of Geodynamics of the Earth's Interior of Moscow State Mining University, Moscow 119991, Russia

## Abstract

Coal seams ascending mining technology is very significant, since it influences the safety production and the liberation of dull coal, speeds up the construction of energy, improves the stability of stope, and reduces or avoids deep hard rock mining induced mine disaster. Combined with the Xiaoming ascending mining mine 4-1, by numerical calculation, the paper analyses ascending mining 4-1 factors, determines the feasibility of ascending mining 4-1 coalbed, and proposes roadway layout program about working face, which has broad economic and social benefits.

## 1. Introduction

Over the years, coal mining follows the principle which is from top to bottom [1–5]. With the exploitation of strength, depth, and scope increasing and complexity of mining conditions, people gradually realize that, in certain geological and mining conditions, the ascending mining has many advantages [6–8]. So mining technology has been widely appreciated. Coal mining in the upstream and abroad gained a wealth of practical experience and scientific research [9–12].

Combined with Xiaoming mine 4-1 upward mining of coal seam, through theoretical analysis and FLAC3D numerical calculation method, this paper analyses the upward mining strata movement rule, abutment pressure distribution, and the working stability [13–16] and then employs reasonable mining program to control and adjust the mining environment in order to guarantee coal mine safety production and both high yield and efficiency [17–20].

## 2. Study Area Summarize

Xiaoming mining is in the late Mesozoic Jurassic coal seam, south third district 408 working face of the 4-1 coal seam lies on the top of south third 708 working face of the underlying 7 coal seam, and the south third 708 working face has been finished. Working face is the south third district with eight parts and seven layers, the dip is 682 m, the width is 167 m, and the area is 107047 m^2^. F316 fault is in the north of the working face, no mining area is in the west, the south third 709 mined areas are in the east, and the F406 fault is in the south, near the Dalong mine. The distance between 7 coal seam and 4-1 coal seam is about 50~54 m, an average of 52 m. The distance between 7 coal seam and 8 coal seam is about 10.82~11.67 m, an average of 11.25 m.

## 3. Theoretical Analysis of 4-1 Coal Seam Ascending Mining

According to the ratio method and the analysis of the “three-zone method,” because the distance between 7 coal seam and 4-1 coal seam is longer (53~57 m), it satisfies the conditions of upstream mining. According to the balance surrounding rock method, south third district 412 working face of the 4-1 coal seam lies on the top of the balance surrounding rock of the underlying working face, and it can be judged to process ascending mining. According to the time interval, south third district 412 working face of the 4-1 coal seam meets the time needed, so it can process ascending mining.

## 4. Numerical Calculation of 4-1 Coal Seam Ascending Mining

### 4.1. Numerical Calculation Model

**Figure 1:**
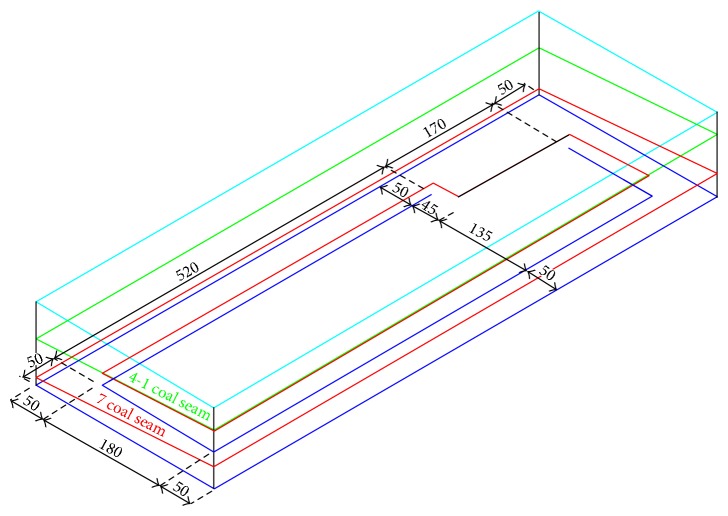
Numerical calculation model of south third 408 working face.

Calculating south third region 708 mined working face of Xiaoming mine by using the software of Flac3D, based on the stress state of overlying rock and deformation characteristics, and analysing the mining effect relationship between 4-1 coal seam and 7 coal seam confirm the feasibility of 4-1 coal seam ascending mining. Establish south third 708 working face model and calculate, according to the geological condition and coal rock condition of the working face (Figure 1). Model range is 790 m (length) × 280 m (width) × 110 m (high), 380 464 meshes and 397 760 nodes were established, the top of the model is −330 m level, and the bottom is −405 m level. Through the simulation of south third 708 working face, it analyses the stress state and distance characteristic of the top coal seam, after south third 708 working face was mined.

### 4.2. Numerical Calculation Analysis

#### 4.2.1. The Stress State of South Third 708 Mined Working Face Surrounding Rock

**Figure 2:**
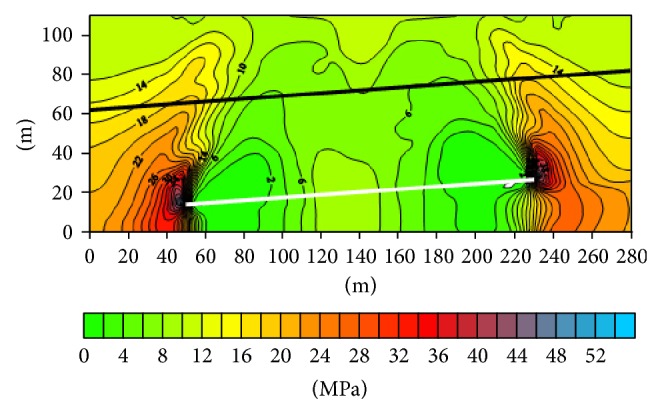
The vertical stress profile along the working face direction.

**Figure 3:**
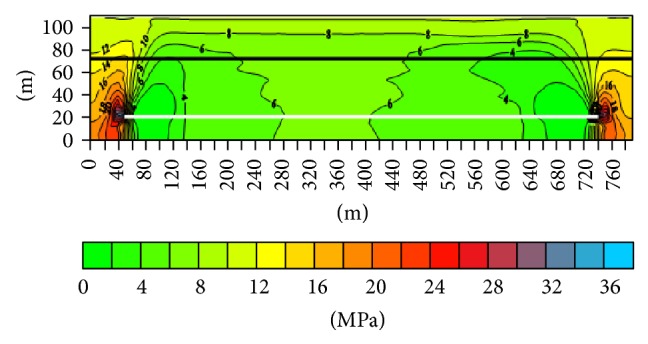
The surrounding rock stress profile along the working face advancing direction.

(1) The vertical stress distribution along the working face advancing direction after being mined (Figure 2): when south third 708 working face is mined, the stress significantly increasing area appears on the boundary of goaf, and the value reaches more than 40 Mpa. The rock of 40~45 m range above 7 coal seam belongs to stress increasing region. The stress decreasing region is on the top of the goaf, and the value reaches 6 Mpa, just half of the normal value. As 4-1 coal seam is subject to 7 coal seam mining effect, the region stress lying in the middle of the goaf decreases, and the stress of both sides increases. Generally speaking, 4-1 coal seam did not show significant increase areas of stress, mainly reflected by the lower impact of mining effects arising from pressure relief.

(2) The surrounding rock stress distribution along the working face advancing direction after being mined (Figure 2): the vertical stress of 4-1 coal seam may only increase in the corresponding position of the lower goaf boundary, and the stress of other regions decreases to 6 Mpa. As the distance between the 4-1 coal seam and 7 coal seam is longer, the effect of 7 coal seam mining coal on 4-1 coal seam is mainly embodied in the influence of stress release of 4-1 coal seam after 7 coal seam is mined. This is for 4-1 coal seam in the process of mining coal seam face strata control and roadway stability has a positive effect. 4-1 coal seam stress increasing region which is along the working face advancing direction is in the lower part of the inner gob 6~8 m (Figure 3).

#### 4.2.2. Shift Characteristic of 4-1 Coal Seam after South Third 708 Working Face Mining

**Figure 4:**
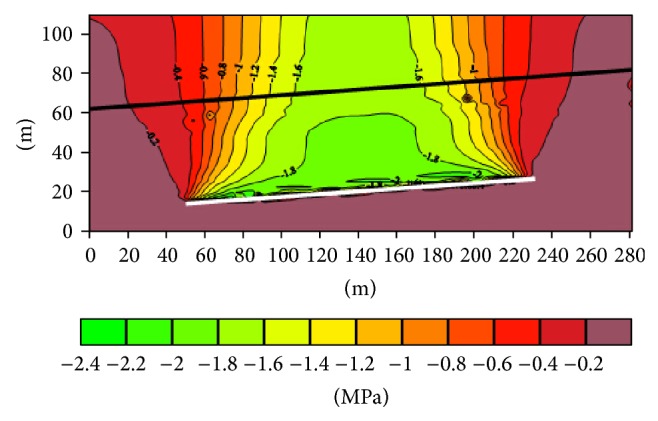
The vertical shift profile along the working face direction.

**Figure 5:**
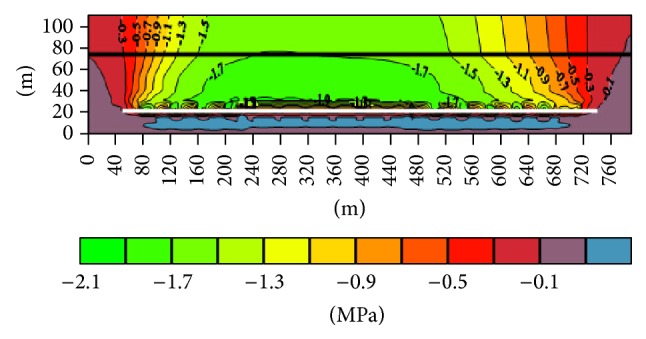
The surrounding rock vertical shift profile along the working face advancing direction.

(1) The distance along the trend of the top 4-1 coal seam after south third 708 working face mining (Figure 4): when the length of the bottom working face is 180 m, the distance maximum of 4-1 coal seam reaches 1.8 m. Subsidence of overlying rock mined mainly appears on the top of the gob, but that of both sides is very small.

(2) Along the advancing direction of displacement of surrounding rock face (Figure 5): after mining of south third 708 working face of 7 coal seam, the subsidence maximum of 4-1 coal seam reaches 1.8 m. The coal seam dip angle is about 1.8°. Therefore, 4-1 coal seam dip angle change is very small, and the continuity of coal has not been significantly affected, still remaining relatively flat state.

After south third 708 working face is mined, the upper 4-1 coal is mined in the lower boundary of the corresponding regional impact of mining on the small range of stress increase, and stress rising value is less, about 1.2~1.4 times of the normal value. The corresponding region of 4-1 coal seam in the lower part of the central stress decreases to 4~6 MPa, and it shows that the lower part of the upper coal seam mining is on a significant role in relief. Overall, as the distance between 4-1 coal seam and 7 coal seam is about 55 m, the main manifestation in which 7 coal seam mining affects the stress state of 4-1 coal seam is stress relief, and therefore the implementation of the south third 408 working face ascending mining is feasible. From the perspective of stress, the lower 7 coal seam mining process for 4-1 coal seam in the face pressure control and the roadway has a positive role in maintaining stability.

## 5. Conclusions

Coal seam ascending mining is of great significance to the mine safety production, improving the recovery rate, improving mine production, extending the service life of mine, improving the stability of stope, and reducing or avoiding the hard rock mining induced deep mine disaster, which has prominent economic and social benefits.
